# Zinc Deficiency as a General Feature of Cancer: a Review of the Literature

**DOI:** 10.1007/s12011-023-03818-6

**Published:** 2023-09-02

**Authors:** Rie Sugimoto, Lingaku Lee, Yuki Tanaka, Yusuke Morita, Masayuki Hijioka, Terumasa Hisano, Masayuki Furukawa

**Affiliations:** Department of Hepato-Biliary-Pancreatology, National Hospital Organization Kyushu Cancer Center, 3-1-1 Notame, Minami-Ku, Fukuoka, 811-1395 Japan

**Keywords:** Zinc deficiency, Trace elements, Cancer diagnosis, Cancer prognosis, Biomarker

## Abstract

Trace elements are minerals that are present in very low concentrations in the human body and yet are crucial for a wide range of physiological functions. Zinc, the second most abundant trace element, is obtained primarily from the diet. After being taken up in the intestine, zinc is distributed to various target organs, where it plays key roles in processes such as immunity, protein folding, apoptosis, and antioxidant activity. Given the important role of zinc in a wide range of enzymatic reactions and physiological processes, zinc deficiency has been identified in a variety of diseases, notably cancer. In recent years, multiple meta-analyses and reviews looking at zinc levels in individual cancer types have been published, as have a plethora of primary studies demonstrating a link between low zinc levels and specific types of cancer. In this review, we summarize recent evidence implicating low zinc concentrations in serum or tissues as a characteristic in a wide range of cancers. We also discuss preliminary findings indicating that zinc level measurement could ultimately become a useful clinical tool for cancer diagnosis and predicting outcomes in patients with cancer. Finally, we suggest future directions for further elucidating the role of zinc deficiency in cancer development and progression.

## Introduction

Trace elements are minerals that are present at very low concentrations in the human body and are crucial for proper physiological function. Some trace elements are considered to be nutritionally essential, meaning they must be obtained from the diet, while others are nonessential. Each trace element is typically present at a concentration of < 0.1% of the body volume and can be readily detected in the blood and other bodily tissues. Trace elements that are well known for their roles in human health and physiology include iron, zinc, iodine, fluoride, and copper. Zinc is the second most abundant trace element after iron [1, 2]. Zinc is obtained primarily from the diet and absorbed in the intestine [3, 4]. Its bioavailability is strongly affected by dietary composition, which affects its uptake by the digestive system [5, 6]. After being taken up in the intestine, zinc is distributed to various target organs throughout the body, with concentrations tending to be highest in bone and muscle, followed by the skin and liver [7].

**Fig. 1:**
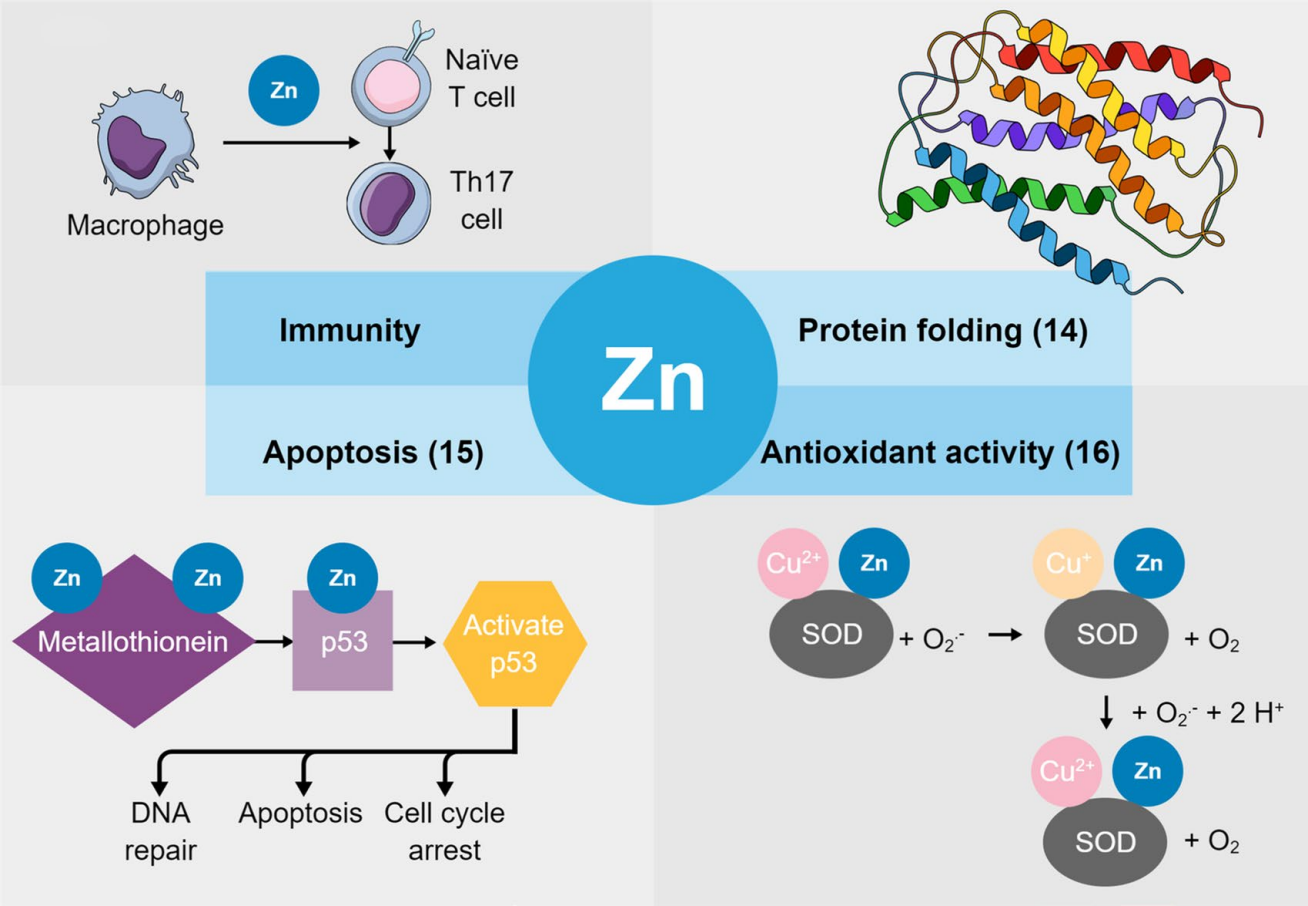
The roles of zinc in human physiology. Some of the most important functions of zinc in the human body include supporting a healthy immune response by promoting differentiation of naïve T cells into activated Th17 cells; facilitating proper folding and activity of zinc-containing proteins; acting as a cofactor for superoxide dismutase (SOD) to exert antioxidant activity; and modulating the stability and activity of p53 to regulate apoptosis. *SOD*, *superoxide dismutase*; *Th17*, *T helper 17.* References: “Protein folding” representative image from [14] CC BY 4.0. “Apoptosis” representative image from [15] CC BY-NC 4.0. “Antioxidant activity” representative image from [16] CC BY 3.0

Zinc plays a variety of key roles in the human body (Fig. 1). For example, it is crucial to signaling processes involved in cell differentiation, proliferation, and apoptosis [8]. Zinc ions are required for the catalytic activity of a multitude of enzymes, notably the zinc finger proteins, which contain a zinc finger motif stabilized by a zinc ion. This large family of proteins has been implicated in processes as diverse as transcriptional regulation, ubiquitin-mediated protein degradation, signal transduction, actin targeting, and DNA repair [9]. In addition to these roles, zinc functions as an antioxidant and plays an essential role in immunity. As reviewed by Prasad, zinc inhibits inflammatory cytokine production and decreases plasma oxidative stress markers [10, 11]. Other studies showed that copper-zinc superoxide dismutase protects tissues from reactive oxygen species, and alterations in the activity of this enzyme have been associated with a wide range of diseases [12, 13].

Unsurprisingly, given the crucial nature of zinc in many enzymatic reactions and physiological processes, low zinc levels are associated with a variety of disease states. Low levels of zinc have been reported in a range of cancers, with increasing evidence suggesting that low zinc levels in serum, plasma, and tissues may be linked to carcinogenesis [17, 18]. In this review, we will present the recent evidence that low zinc levels in a variety of situations are a hallmark of various cancers. We will also speculate on the usefulness of zinc level as a biomarker and the potential prevention or treatment of cancer by zinc supplementation, as reported in preliminary studies. Finally, we will highlight the research priorities for increasing the understanding of the role that zinc deficiency plays in cancer and developing potential clinical applications on the basis of this association in the future.

### Zinc Deficiency in Cancer

In this section, we provide a comprehensive review of the current state of knowledge regarding zinc levels in patients with various cancer types. Table 1 presents an overview of the key features of the studies discussed in this section. We used PubMed to identify 2470 articles on cancer and zinc published up to 2023. We excluded case reports, articles on animals, articles on side effects occurring during and after cancer treatment, articles in which the carcinoma was not specified, and basic research, resulting in a total of 151 articles. Clinical/epidemiological articles were further selected from these studies, and we excluded review articles; articles with the highest number of cases, highest level of evidence, and newest articles by cancer type were selected and are listed in Table 1.

**Table 1:** Clinical/epidemiological publications up to 2023 reporting a link between low zinc levels and cancer

*Cancer type*	*Study type*	*Number of study participants*	*Reference*
*Esophageal cancer*	Case–control study	218 cases, 415 controls	Lu et al. (2006) [19]
	Ecological analysis	32 countries	Schaafsma et al. (2015) [20]
	Prospective study	47,405 subjects 201 patients	Hashemian et al. (2015) [21]
	Cross-sectional studies	30 patients, 30 controls 30 patients, 30 controls	Ray et al. (2012) [22]
*Breast cancer*	Cross-sectional age-matched controlled study	30 patients, 30 controls	Adeoti et al. (2015) [23]
	Retrospective in situ study of patient samples	25 patients	Costello et al. (2016) [24]
	Meta-analysis	926 patients, 1224 controls	Wu et al. (2015) [25]
	Meta-analysis	1699 patients, 2009 controls	Jouyabari et al. (2019) [26]
	Meta-analysis	2369 patients with breast cancer, 901 patients with benign breast disease, 2477 healthy controls	Feng et al. (2020) [27]
	Prospective case–control study	496 patients, 496 controls	Pala et al. (2022) [17]
	Prospective study	63 patients	Mansouri et al. (2022) [28]
	Prospective study	40 patients	Baratabar et al. (2022) [29]
Liver cancer	Case–control studies	37 patients, 33 controls	Kew et al. (1974) [30]
	Retrospective in situ study of patient samples	51 patients	Tashiro-Ito et al. (1997) [31]
	Prospective study	175 patients	Tamai et al. (2020) [32]
	Retrospective study	310 patients	Imai et al. (2014) [33]
	Prospective cohort study	989 patients	Fang et al. (2019) [34]
	Prospective study	157 patients	Shigefuku et al. (2020) [35]
	Retrospective study	769 patients	Ozeki, Nakajima et al. (2020) [36]
	Prospective study	1973 patients	Ozeki, Arakawa et al. (2020) [37]
	Retrospective study	196 treated patients, 71 untreated patients	Hosui et al. (2018) [38]
	Retrospective study	599 patients	Hosui et al. (2021) [18]
*Lung cancer*	Meta-analysis	2894 cases, 9419 controls	Wang et al. (2019) [39]
	Systematic review and meta-analysis	3598 lung cancer patients, 1402 benign lung disease cases, 3314 healthy controls	Zhang et al. (2022) [40]
	Case–control study	440 cases, 1320 controls	Bai et al. (2019) [41]
Gynecological cancer	Meta-analysis	591 patients, 946 controls	Xie et al. (2018) [42]
	Meta-analysis	699 patients, 567 benign tumor, 1194 controls	Lin et al. (2021) [43]
Colon cancer	Case–control study	966 cases, 966 controls	Stepien et al. (2017) [44]
	Prospective observational study	116 patients	Wu et al. (2020) [45]
Oral cancer	Case–control study	344 patients, 1122 controls	Chen et al. (2019) [46]
	Case–control study	463 cases, 1343 controls	Wang et al. (2020) [47]
Other cancers	Systematic review and meta-analysis (prostate cancer)	1318 patients, 1413 controls	Zhao et al. (2016) [48]
	Prospective study (pancreatico-biliary cancer)	48 patients	Murphy et al. (2021) [49]
	Longitudinal cohort study (pediatric hematologic and solid malignancies)	535 patients	Ganguly et al. (2022) [50]
	Prospective cohort study (acute myeloid leukemia)	105 patients, 82 controls	Li et al. (2023) [51]

### Zinc Deficiency in Esophageal Cancer

Esophageal cancer is one of the cancer types that is the most well studied in relation to zinc and cancer incidence. Lu et al. [19] reported in 2006 that the zinc intake of esophageal cancer patients in China was lower than that of healthy individuals, and Schaafsma et al. [20] conducted a database-based ecological analysis and reported that in Africa, zinc supply was lower in countries with a high risk for esophageal cancer incidence than in low-risk countries. In 2015, a significant negative correlation between zinc intake and the incidence of esophageal cancer was reported in the Golestan cohort study in a high-risk district for esophageal cancer [21]. Zinc levels in hair were measured in high- and low-risk areas for esophageal cancer, and low zinc levels were found to be associated with the development of esophageal cancer [22]. The molecular mechanisms linking esophageal cancer and zinc deficiency are also being elucidated, with a large body of evidence indicating that zinc deficiency promotes carcinogenesis by altering microRNA expression [52–54]. Li et al. [55] reported a significant correlation between zinc and risk of esophageal cancer in Asia, where zinc intake is naturally low, but no correlation was found in the USA or Europe. Dietary zinc intake also varies widely from region to region because of the strong regional specificity of dietary habits. When discussing the relationship between dietary zinc intake and disease, it may be necessary to examine each region separately.

### Zinc Deficiency in Breast Cancer

There is considerable evidence for an association between zinc levels in tissue and serum and breast cancer, as demonstrated by many recent studies of zinc levels in a variety of patient tissues.

Early prospective studies provided clear indications that low zinc levels are closely linked to breast cancer development and progression. In their 2015 cross-sectional study conducted in Nigeria, Adeoti et al. [23] reported that the copper/zinc ratio was significantly higher in patients with breast cancer compared with healthy controls and that venous blood zinc levels were low in breast cancer patients. A retrospective in situ study by Costello et al., published in 2016, reported low zinc levels in breast invasive ductal carcinoma compared with normal ductal epithelium [24].

Several meta-analyses were published that collated evidence regarding zinc deficiency in breast cancer. A 2015 meta-analysis by Wu et al. [25] assessed zinc levels in various tissue samples from female breast cancer patients and healthy controls. The authors found that zinc levels in hair samples from breast cancer patients were lower than those in hair samples from a control group; however, they reported no significant difference in serum zinc levels between these two groups. More recently, a 2019 meta-analysis investigated zinc levels in breast tissue, plasma, serum, and hair samples from patients with breast cancer [26]. The authors reported that zinc levels were significantly lower in blood and hair samples from patients with breast cancer compared with controls, and significantly higher in tumor tissues. Furthermore, a 2020 meta-analysis by Feng et al. [27] evaluated data from studies assessing serum copper and zinc levels in patients with breast cancer. The results showed that elevated serum copper levels, elevated serum copper/zinc ratio, and decreased serum zinc levels were significantly associated with an increased risk of breast cancer. It should be noted, however, that in these meta-analyses, there was overlap in the cohorts used, as the hair sample cohort in the 2015 and 2019 meta-analyses was identical [25, 26]. Furthermore, among the cohorts used in the 2019 and 2020 meta-analyses, half of the cohorts examined for serum zinc were the same (duplicates) [26, 27].

Since the publication of these meta-analyses, additional clinical studies have shed further light on the close association between low zinc levels in tissues and serum and breast cancer. For example, in their 2022 study, Pala et al. [17] assessed the association between pre-diagnostic copper and zinc levels in the plasma and urine of women later diagnosed with breast cancer. The authors found that patients with a plasma copper/zinc ratio or a plasma and urine copper/zinc ratio in the upper tertile of the patient cohort had a significantly higher risk of developing breast cancer than those in the lower tertiles. A 2022 study by Mansouri et al. [28] reported that higher zinc levels were present in mammary tissue from healthy individuals than in breast cancer tissue from patients. In contrast, in a 2022 study of breast cancer patients, Barartabar et al. [29] reported lower zinc levels in adjacent non-cancerous areas compared with breast cancer tissue. This apparent discrepancy should be interpreted with caution, as Mansouri et al. assessed zinc levels in tissue from breast cancer patients compared with healthy controls, while Barartabar et al. examined zinc levels in healthy and diseased tissue from the same patient; thus, the studies may not be comparable.

Taken together, these meta-analyses and clinical studies build on the existing evidence for a correlation between breast cancer and zinc deficiency.

### Zinc Deficiency in Liver Cancer

In addition to breast cancer, there is a substantial body of evidence demonstrating an association between low zinc levels and liver cancer.

Many studies have shown low zinc concentrations in liver cancer tissue. Kew et al. [30] reported that zinc concentrations in liver cancer tissue were significantly lower than those in non-cancerous areas, and further reports showed that zinc concentrations decrease as the degree of differentiation of liver cancer worsens [31]. In 2014, Costello et al. [56] highlighted the relationship between zinc levels and chronic liver disease/hepatocarcinogenesis, citing numerous studies that reported a marked decrease in zinc levels in hepatocellular carcinoma tissue compared with normal liver tissue. At present, no published papers have reported results indicating that zinc levels are not decreased in hepatocellular carcinoma tissue. Another study reported that serum zinc levels decrease as hepatocellular carcinoma progresses [32].

Low zinc levels also appear to correlate with poor outcomes in patients treated for liver cancer. For example, a retrospective analysis by Imai et al. [33] published in 2014 found that low serum zinc levels correlate with low disease-free and overall survival rates in patients undergoing hepatectomy for hepatocellular carcinoma. Interestingly, a 2019 study by Fang et al. [34] found that a higher serum copper/zinc ratio, but not a higher serum zinc level, may be associated with survival in patients with hepatocellular carcinoma. However, the actual risk of death in patients with low zinc levels in the study was not clear, as the participants were stratified into multiple groups with a range of zinc levels, and survival rates were compared among all of the groups. As copper and zinc absorption are antagonistic processes, more research is needed to determine whether copper or zinc plays a role in the prognosis of patients with liver cancer. Additionally, Hiraoka et al. reported that patients with low serum zinc levels had a worse prognosis than those with high levels, but there was no difference in recurrence-free survival. Furthermore, in the analysis of prognosis after treatment of early liver cancer, zinc levels correlated with hepatic reserve capacity [57]. Thus, the relationship between zinc and the prognosis of liver cancer is not only related to the prognosis of the cancer itself, but also to the prognosis related to hepatic reserve capacity.

Zinc deficiency appears to play a role not only in liver cancer outcomes but also in the risk of liver cancer development. Shigefuku et al. [35] prospectively observed 157 cirrhotic patients for 3 years and reported that hypozincemia was the only significant predictor of hepatic carcinogenesis. In 2020, Ozeki et al. [36] reported that low zinc levels were a risk factor for developing hepatocellular carcinoma after eradication of hepatitis C virus (HCV). Another report from Ozeki et al. [37] published in the same year found that low zinc levels are highly prevalent in patients with chronic liver disease, a strong risk factor for developing hepatocellular carcinoma. In considering the possible link between hepatocarcinogenesis and zinc, it is interesting to note that Franklin et al. [58] reported that tissue zinc deficiency is present in highly differentiated hepatomas and observed alongside downregulation of the ZIP14 zinc uptake transporters. This suggests that zinc depletion begins in the precancerous state. The connection between low zinc levels and hepatocarcinogenesis was explored in a 2022 review by Nishikawa et al.; the authors discussed a variety of studies demonstrating the relationship between hypozincemia and chronic liver disease, liver fibrosis, and the risk of liver carcinogenesis [59]. Whether hypozincemia is really the direct cause of hepatocarcinogenesis or whether hypozincemia correlates with liver fibrosis and only results in increased hepatocarcinogenesis remains unclear, and further research is needed.

Recent studies have explored the impact of zinc supplementation on the risk of developing liver cancer. A 2018 retrospective study by Hosui et al. [38] found that patients with chronic liver disease who received long-term zinc supplementation had a lower risk of developing liver cancer than patients who did not take the supplement. In 2021, the same group reported that supplementation with zinc after HCV eradication reduces the risk of developing hepatocellular carcinoma [18].

Together, these studies provide a strong argument for a key role for zinc in liver cancer development and progression.

### Zinc Deficiency in Lung Cancer

Multiple recent studies support a link between zinc deficiency and lung cancer, and the association was extensively analyzed in two recent meta-analyses. Wang et al. found that serum zinc levels were significantly lower in patients with lung cancer than in controls in European and Asian populations [39]. Similarly, Zhang et al. found that lung cancer patients had a significantly higher serum copper/zinc ratio (implying low zinc levels) than healthy controls and patients with benign lung diseases; this ratio was significantly higher in patients with advanced lung cancer than in patients with early-stage lung cancer [40]. Notably, the two meta-analyses included 32 and 39 cohorts, but the results were not derived from completely separate populations because of the overlap of 23 cohorts. Additionally, Bai et al. reported that high zinc plasma reduces the risk of lung cancer, possibly by delaying telomere events and modulating the expression of some oncogenes; the authors suggest the possibility that zinc can be used to prevent lung cancer [41]. These studies clearly show that zinc deficiency is a factor in lung cancer development and progression.

### Zinc Deficiency in Gynecological Cancer

Zinc deficiency has been well described in gynecological cancers.

The association between low zinc levels and cervical cancer was convincingly demonstrated in a 2018 meta-analysis by Xie et al. [42], which reported significantly lower serum zinc levels in cervical cancer cases than in controls in Asian women.

In endometrial cancer, reports on the relationship between cancer and zinc concentrations are mixed. Yaman et al. [60] reported that zinc concentrations in cancerous tissues were significantly lower than those in non-cancerous tissues, while Rzymski et al. [61] found no difference in zinc levels in cancerous areas despite an increased copper/zinc ratio. In contrast, Atakul et al. [62] reported lower serum copper and zinc levels but lower copper/zinc ratios in patients with endometrial cancer. Further study on the association between zinc concentration and endometrial cancer will be required.

Lin et al. reported a meta-analysis and Mendelian randomization study in ovarian cancer in 2021. The authors showed that ovarian cancer patients have significantly lower circulating zinc concentrations compared with healthy individuals [43].

Overall, the evidence strongly suggests that low zinc levels are associated with a range of gynecological cancers, and that an interaction with copper levels may also play a role. It should be noted that there is, as yet, no evidence that low zinc levels are a cause of carcinogenesis or cancer progression in gynecological cancers, and at this point, the results have only suggested a possible relationship between zinc levels and cancer patients.

### Zinc Deficiency in Colon Cancer

Colon cancer has also been reported to be associated with zinc deficiency. A 2017 case–control study by Stepien et al. [44] performed using the large European Prospective Investigation into Cancer and Nutrition cohort reported an apparent link between copper/zinc ratios and colorectal cancer. More recently, a 2020 prospective observational study reported a correlation between low serum zinc levels and poor prognosis (including distant metastasis and survival) in patients with colon cancer [45]. While this association has not been explored as extensively as it has for other cancer types, the findings from these two studies suggest that zinc deficiency is also associated with colon cancer incidence and outcomes. However, while a Mendelian randomized study of 58221 European patients with colon cancer reported that a nominally significant association between colon cancer risk and zinc concentration was found, sensitivity analysis could not be performed, and ultimately a causal relationship with risk could not be proven [63]. Only a few mouse studies have examined whether zinc deficiency causes colon cancer [64]. Thus, more research is needed to determine whether zinc deficiency causes colon cancer.

### Zinc Level in Oral Cancer

Notably, the association between zinc concentration and carcinogenesis in oral cancer is different from that of other carcinomas. Two recent case–control studies revealed a link between zinc levels and oral cancer. A 2019 study by Chen et al. [46] found that both excess and deficient serum levels of copper or zinc are correlated with oral cancer risk. A large 2022 case–control study by Wang et al. [65] assessed the association between oral cancer and a range of trace elements and reported that higher serum zinc levels correlated with a higher risk of oral cancer. Other studies reported that serum zinc levels [66] and salivary zinc levels [67] are higher in cancer patients than in healthy individuals. Whether the difference from other cancer types is because the oral mucosa is the site of direct contact with food and is susceptible to high concentrations of zinc, or whether it is from other causes, requires further investigation.

### Zinc Deficiency in Other Cancers

In addition to the specific cancers covered in detail above, zinc deficiency has been reported in a range of other cancers. In 2016, a systematic review and meta-analysis by Zhao et al. [48] reported that serum zinc concentrations were significantly lower in patients with prostate cancer than in those with benign prostatic hyperplasia and in normal controls. Notably, however, high doses of zinc supplementation were reported to be a risk factor for prostate cancer [68]. More recently, a 2021 study from the UK by Murphy et al. [49] looked at micronutrient levels in patients with pancreatico-biliary cancer and identified low levels of serum zinc in 83% of the study cohort. A 2022 cohort study by Ganguly et al. [50] conducted in a cancer center in India in children aged ≤ 18 years found that zinc deficiency correlated with poor outcomes in patients with solid tumors. A 2023 prospective cohort study by Li et al. found that a high serum copper/zinc ratio was associated with poor outcomes and long-term survival in patients with newly diagnosed acute myeloid leukemia [51]. While these studies have not shown zinc deficiency to be a direct cause of cancer, they do provide clear preliminary evidence of an association between zinc deficiency and various types of cancer.

## Potential Clinical Utility of Measuring Zinc Levels in Cancer

Given the substantial evidence for zinc deficiency as a universal feature of cancer that is common in a relatively large number of cancers, it seems likely that measuring zinc levels could become useful in the clinical setting in the near future. While this is still an emerging area of research, early indications suggest that zinc level measurement could potentially be used to diagnose cancer, predict cancer patient outcomes, and even treat patients with cancer. In this section, we discuss the preliminary evidence for these speculative applications.

### Zinc as a Potential Diagnostic Biomarker for Cancer

Multiple studies have suggested that circulating zinc levels could be used as a diagnostic biomarker for cancer in patients suspected of having or being at risk of developing a malignancy. For example, a 2022 review by Venturelli et al. [69] explored the associations between cancer and minerals and trace elements. Among other factors, the authors noted a direct link between zinc levels and the development of different cancer types, suggesting that this micronutrient has potential for use as a biomarker for cancer, while highlighting the interdependence of micronutrient levels. Additionally, a 2017 review by Lo et al. [70] highlighted the low zinc levels found in prostate cancer tissues compared with healthy prostate tissue and recommended the use of zinc-responsive magnetic resonance imaging as a noninvasive means of diagnosing prostate cancer. Similarly, a prospective study by Maddalone et al. published in 2022 analyzed zinc levels in urine samples collected after prostatic massage from men referred for prostate biopsy; the results found that zinc levels were lower in the urine of cancer patients than in healthy subjects and decreased with disease progression [71]. The authors therefore suggested that urine zinc levels could be combined with other diagnostic modalities to identify patients with prostate cancer. The aforementioned 2020 study by Ozeki et al. [36] found that low serum zinc levels correlated with an increased risk of developing hepatocellular carcinoma following HCV eradication with direct-acting antivirals. The authors suggested that monitoring serum zinc levels in this specific patient population could enable early diagnosis of this malignancy. Thus, while the study of zinc as a potential biomarker for diagnosing a range of cancer types is still in its infancy, it is an idea that seems to be not only plausible but also highly applicable to specific patient populations. However, it is extremely important to note that serum zinc levels fluctuate under a variety of conditions. There are diurnal variations in zinc concentrations [72], and zinc levels also vary on the basis of sepsis [73], endotoxemia from alcohol consumption [74], steroid use [75], and mealtimes [76]. Michos et al. [77] also reported that zinc levels fluctuate on the basis of the menstrual cycle. These conditions need to be considered before zinc level is considered a biomarker for cancer.

### Zinc as a Potential Prognostic Biomarker for Cancer

Zinc levels have also been proposed as a prognostic biomarker for predicting outcomes in patients with cancer. For example, a recent study by Iseki et al. [78] indicated an association between serum zinc status and postsurgical outcomes in patients with pancreatic ductal adenocarcinoma. Specifically, infectious complications were significantly associated with zinc deficiency in patients undergoing resection for this malignancy, suggesting that zinc deficiency could serve as a preoperative predictor of infectious complications after pancreatectomy in these patients. Gal et al. [79] also reported that, in patients undergoing exploratory laparotomy for suspected ovarian cancer, copper and zinc ratios, in addition to CA125, were sensitive in predicting ovarian cancer before laparotomy. Similarly, Harimoto et al. [80] reported in 2022 that serum zinc status may correlate with a range of outcomes in patients undergoing hepatic resection for hepatocellular carcinoma. Thus, zinc levels in these patients could be screened to predict which patients are at risk of suffering worse liver function, more severe liver fibrosis, a higher incidence of postoperative complications, and worse overall survival. Furthermore, a 2020 study by Tamai et al. [32] showed that a higher copper-to-zinc ratio in serum was associated with significantly improved survival rates in patients with hepatocellular carcinoma, indicating that the copper-to-zinc ratio in serum may be used to predict patient survival. A 2020 study by Hiraoka et al. [57] reported that in patients with early hepatocellular carcinoma from hepatitis virus, serum zinc levels were reduced in association with chronic liver disease grade progression, suggesting that zinc deficiency might be a significant prognostic factor for survival in this patient population. Thus, established links between zinc deficiency and patient outcomes could support the use of zinc level measurement as a useful tool for predicting cancer patient prognoses in the future. Notably, there are still many points to consider, such as the timing of measurement, zinc fluctuations because of background factors, and changes in zinc concentration due to complications.

### Zinc as a Potential Preventive Method for Cancer

On the basis of the correlation between low zinc levels and poor cancer outcomes, many authors have speculated that zinc supplementation could be used to reduce the risk of developing cancer. A retrospective analysis published by Hosui et al. [38] in 2018 reported that zinc supplementation appears to maintain liver function and decrease the risk of developing hepatocellular carcinoma. A more recent study by the same group found that oral zinc supplementation decreased the risk of hepatocellular carcinoma development in patients who received direct-acting antivirals to eradicate HCV [18]. Valenzano et al. [81] also reported that in Barrett’s esophagus, administration of zinc gluconate resulted in the upregulation of several tumor-suppressive miRNAs and downregulation of inflammation-inducing proteins. Additionally, a 2022 mini-review by Iqbal et al. [82] noted limited evidence for a correlation between high dietary intake of zinc and a reduced risk of breast cancer, suggesting that dietary supplementation could decrease the chance of developing this malignancy. These preliminary studies suggest that there could be potential for using zinc supplementation in the clinical setting to help prevent cancer development.

Zinc supplementation has also been suggested by several studies as a potential adjunctive treatment for cancer. A series of papers by Costello et al. [56] directly addresses this possibility: a 2014 review by this group suggests that zinc supplementation could be a useful treatment for hepatocellular carcinoma, while also pointing out the limitations of existing in vitro models that have made it challenging to generate pre-clinical evidence for the utility of this therapeutic approach; a 2017 review presents compelling evidence that zinc treatment could help prevent the development and progression of prostate, liver, and pancreatic carcinomas [83]; and a 2020 review again points out the striking correlation between zinc deficiency and multiple cancer types and cites a single case report that demonstrated suppression of androgen-dependent prostate cancer progression in a patient treated with the zinc ionophore clioquinol [84]. Additionally, in a review published in 2020, Wang et al. [47] highlighted the prevalence of zinc dyshomeostasis in prostate cancer, breast cancer, and pancreatic cancer and provided an overview of the current evidence suggesting that zinc or zinc transporters could therefore be useful agents for cancer therapy. Furthermore, a 2021 systematic review by Hoppe et al. [85] that analyzed data from 19 publications found that zinc therapy helped reduce oral toxicities incurred during irradiation in patients mainly diagnosed with head and neck cancer. It should be noted, however, that high doses of zinc supplementation have been reported to be a risk factor for prostate cancer [68]. While a 2021 review by Singh et al. [86] mentions several studies that showed no beneficial effect of zinc supplementation on zinc levels in patients with prostate cancer, the authors proposed combined treatment with zinc and naturally occurring dietary phytochemicals that could lead to enhanced zinc bioaccumulation in the prostate. A 2022 review by Nishikawa et al. [59] describes the recent approval of zinc acetate hydrate for treating patients with hypozincemia associated with chronic liver disease in Japan and presents evidence from several studies showing a positive effect of zinc supplementation in patients with various forms of liver disease putting them at risk of developing liver cancer. Together, these studies have highlighted promising preliminary indications that zinc supplementation could be a useful addition to cancer treatment regimens and identified specific areas for future investigation.

## Conclusion

The extensive evidence demonstrating the prevalence of zinc deficiency in a wide range of cancer types suggests that zinc deficiency should be considered a relatively widespread feature of multiple cancers. While research regarding the potential clinical utility of testing zinc levels in patients with or at risk of developing cancer is still preliminary, the data suggest that zinc deficiency may be a potential biomarker for identifying patients at risk of developing cancer, predicting outcomes in patients with cancer, and even as a preventive or adjunctive treatment for cancer.

There are several avenues for future research regarding the link between cancer and zinc deficiency that would be valuable to pursue. At a basic research level, future work should focus on elucidating the mechanisms by which zinc deficiency promotes carcinogenesis. Clinically, it would be beneficial to explore the relationship between serum zinc levels and local zinc concentrations in patients with cancer, as well as the reliability of serum zinc levels as a biomarker of carcinogenesis risk and cancer patient prognosis. Finally, prospective clinical studies should be carried out to determine the prognostic benefits of zinc supplementation. Given the universality of zinc deficiency in cancer, gaining a greater understanding of the molecular basis and clinical impact of this association is likely to yield substantial benefits for human health.

## Data Availability

Not applicable.
